# Underdetermined DOA Estimation for Wideband Signals via Focused Atomic Norm Minimization

**DOI:** 10.3390/e22030359

**Published:** 2020-03-20

**Authors:** Juan Shi, Qunfei Zhang, Weijie Tan, Linlin Mao, Lihuan Huang, Wentao Shi

**Affiliations:** 1 School of Marine Science and Technology, Northwestern Polytechnical University, Xi’an 710072, China; shijuan-1029@163.com (J.S.); zhangqf@nwpu.edu.cn (Q.Z.); hyb504@mail.nwpu.edu.cn (L.H.); 2 State Key Laboratory of Public Big Data, Guizhou University, Guiyang 550025, China; tanweijie@hotmail.com; 3 Institute of Acoustics, Chinese Academy of Sciences, Beijing 100190, China; maoll@mail.ioa.ac.cn

**Keywords:** underwater acoustic signal processing, wideband signals, direction of arrival (DOA) estimation, nested array (NA), atomic norm minimization (ANM), information theory criteria

## Abstract

In underwater acoustic signal processing, direction of arrival (DOA) estimation can provide important information for target tracking and localization. To address underdetermined wideband signal processing in underwater passive detection system, this paper proposes a novel underdetermined wideband DOA estimation method equipped with the nested array (NA) using focused atomic norm minimization (ANM), where the signal source number detection is accomplished by information theory criteria. In the proposed DOA estimation method, especially, after vectoring the covariance matrix of each frequency bin, each corresponding obtained vector is focused into the predefined frequency bin by focused matrix. Then, the collected averaged vector is considered as virtual array model, whose steering vector exhibits the Vandermonde structure in terms of the obtained virtual array geometries. Further, the new covariance matrix is recovered based on ANM by semi-definite programming (SDP), which utilizes the information of the Toeplitz structure. Finally, the Root-MUSIC algorithm is applied to estimate the DOAs. Simulation results show that the proposed method outperforms other underdetermined DOA estimation methods based on information theory in term of higher estimation accuracy.

## 1. Introduction

Direction of arrival (DOA) estimation is an important issue in numerous fields of array signal processing, which has widespread applications in radar, sonar, acoustics, navigation, communications, speech enhancement [1,2,3] and multiple-input multiple-output (MIMO) systems [4]. Along with the recent development of underwater sonar systems, DOA estimation could supply the direction information, which plays various important roles in underwater target tracking [5,6,7] or target localization. Currently, plenty of mature and robust DOA estimation methods, such as estimating signal parameter via rotational invariance techniques (ESPRIT), multiple signal classification (MUSIC), etc. have achieved super-resolution estimation performance for narrowband signals [8]. Further, compared with narrowband signal sources, the wideband counterparts are able to provide more useful and interfering information where the array phase differences lie on two factors, i.e., the DOAs of sources and frequencies. Meanwhile, in passive sonar system, the radiation noise signal [9] from underwater vehicles is also a wideband signal. In light of such characteristics, wideband DOA (WDOA) estimation has attracted more research interests [10,11,12]. Among the classical wideband processing algorithms, the incoherent signal subspace method (ISM) can only deal with uncorrelated signal, which averages over the results of all frequency bins as the final fuse result. Alternatively, the coherent signal subspace method (CSM), whose main idea is to collect the averaged array covariance matrix by the focusing technique, not only can handle both uncorrelated and correlated signals, but also improves DOA estimation performance [13].

Traditionally, using a uniform linear array (ULA) formed with *M* sensors can detect at most M−1 signal sources successfully. However, handling underdetermined DOA estimation with fewer physical sensors than signal sources is a fundamental challenge and has great attention and concern in recent years [14,15,16,17,18]. To tackle such wideband DOA estimation issue, the sparse array structure has been proposed as one possible solution. Currently, two classes of recognized sparse linear arrays (SLA), i.e., nest arrays (NA) [19] and coprime arrays (CPA) [20,21], have been utilized to construct a virtual array based on the difference co-array concept theory, which enable to increased number of degrees of freedom (DOF), since the coordinate positions of virtual sensors are established by the consecutive and non-consecutive lag differences among the physical sensors. The CPA can deal with MN signal sources equipped with (M+N+1) sensors. Unfortunately, it fails to apply the augmentation techniques directly because it is unable to construct a filled co-array. By contrast, NA can easily generate a filled difference co-array, which makes itself one of the most attractive array configuration.

Alternatively, compressed sensing (CS), based on sparse signal processing techniques, has worked well in the DOA estimation application [22,23,24], whose irreplaceable advantage is to allow the DOA estimation with deficient snapshots, especially single snapshots, without enlarging the array aperture size [25]. Compared with traditional signal subspace methods, i.e., the spatial smoothing MUSIC, underdetermined CS-based DOA estimation attains not only a higher number of DOF but also better DOA estimation performance owing to the exploitation of all the unique co-array lags in lieu of only the consecutive part. Shen et al. [26] proposed a focused compressive sensing DOA estimation method for the underdetermined wideband signals, which enables reducing the complexity. However, the conventional CS approach has several huge disadvantages: (1) The true signal DOAs are always supposed to fall onto the predefined grid, otherwise the signals cannot be represented by the discrete dictionary [27]. (2) It lies on a finite-size DOAs grid, and finer grids give rise to the numerical instability issue. In particular, the true DOAs are not necessarily assumed to lie on a grid.

Developed as a gridless CS approach, Tang et al. [28] proposed a gridless atomic norm technique, which can be formulated as semidefinite programming (SDP) to recover off-grid sources with high probability from compressive measurements. The ANM approach attains off-grid estimation at super-resolution estimation performance of parameter due to the Vandermonde structure of the signal.

In this paper, aiming at addressing the problem of fewer physical sensors than signals in DOA estimation application, we propose a novel underdetermined DOA estimation method for wideband signals equipped with NA, which is based on ANM to seek and obtain the optimal Toeplitz covariance matrix. Firstly, inspired by SLAs’s benefits, the virtual array formed by the difference co-array is used as a substitute for the physical array geometry. We then build the virtual signal mode through vectorizing the covariance matrix of each frequency bin, and collecting the average vector by focusing technique. Finally, the Toeplitz covariance matrix is recovered via the ANM, which is beneficial for improving the estimation accuracy. The reason is that the retrieved Toeplitz covariance matrix is not only a low-rank Toeplitz matrix, but also a denoising covariance matrix. In addition, we use the information theory methods, named Akaike’s information criterion (AIC), minimum description length (MDL), and corrected AIC (AIC-C3) [29,30], to detect the number of signal sources.

The rest of this paper is organized as follows. In Section 2, the wideband signal model and problem formulation are introduced. The novel underdetermined wideband DOA estimation method via focused ANM is proposed in Section 3. Comprehensive simulations are presented to verify the performance of the proposed novel method in Section 4, followed by the conclusions in Section 5.

**Notations:** Vector **a** and matrices **A** are represented by lowercase and uppercase bold characters, respectively. A refers to an atom set. C and R denote the complex number and the real number, respectively. ${\left(\cdot \right)}^{H}$ and ${\left(\cdot \right)}^{*}$ represent the conjugate transpose and conjugate, respectively. ${∥a∥}_{2}$ is the $\ell_{2}$ norm of a. Symbols ${∥\cdot ∥}_{F}$, Rank(·), and tr(·) stand for the Frobenius norm, rank, and trace of a matrix, respectively. Expectation operator is denoted by E[·]. diag(·) implies that a diagonal matrix is formed from the given vector as the diagonal elements. vec{·} denotes the vectorization operator. ⊗ denotes the Kronecker product and ⊙ refers Khatri–Rao product. ⌊·⌋ represents rounding down.

## 2. Signal Model and Problem Formulation

**Figure 1:**
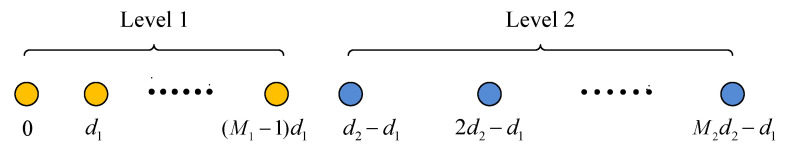
A two-level NA with $M_{1}$ sensors in the inner sub-array and $M_{2}$ sensors in the outer sub-array.

Consider a two-level nested arrays of *M* sensors with the $M_{1}=\left\lfloor \frac{M}{2}\right\rfloor$ sensors in the inner sub-array, while $M_{2}=M-M_{1}$ sensors in the outer sub-array, and both sub-arrays are the uniform linear array (ULA). $d_{1}$ denotes the separation between the sensors of the inner sub-array, while $d_{2}$ represents the counterpart of the outer sub-array, which is equivalent to $\left(M_{1}+1\right)d_{1}$. The NA structure is shown in Figure 1, in which the position of the sensors are given by the sets

(1) $$\begin{array}{cc} H= & \left\{h_{m}:m=1,2,\cdots ,M\right\}\\ = & \left\{m_{1}d_{1},m_{1}=0,1,\cdots ,M_{1}-1\right\}\;\cup \left\{m_{2}d_{2}-d_{1},m_{2}=1,2,\cdots ,M_{2}\right\}. \end{array}$$

Assume that *K* wideband stationary signals at the directions of $\theta_{1},\theta_{2},\ldots ,\theta_{K}$ from the far-field impinge on the nested array, where $\theta_{k}$ represents the *k*th signal source. Then, the received signal $x^{m}\left(t\right)$ of the *m*th sensor can be expressed as

(2) $$x^{m}\left(t\right)=\sum_{k=1}^{K}s^{k}\left(t-\tau_{m}\left(\theta_{k}\right)\right)+w^{m}\left(t\right),m=1,2,\cdots ,M,$$

where the $w^{m}\left(t\right)$ is noise part, whose entries are zero-mean Gaussian random variables with $\sigma^{2}$ variance. $\tau_{m}\left(\theta_{k}\right)$ represents the propagation delay associated with the *k*th signal impinging and the *m*th sensor.

Wideband signal after time-sampling is split into *L* non-overlapping segments, and an *N*-point discrete Fourier transform (DFT) is carried out over each segment data. The output at frequency bin $f_{n}$ is modeled as

(3) $$x_{n,l}=A_{n}\left(\theta \right)s_{n,l}+w_{n,l}\;\;\;n=1,2,\cdots ,N;\;\;l=1,2,\cdots ,L,$$

where $s_{n,l}$ is the signal vector and $w_{n,l}$ represents the additive noise vector in the frequency domain. The array manifold matrix $A_{n}\left(\theta \right)\in C^{M\times K}$ of the *n*th frequency is

(4) $$A_{n}\left(\theta \right)=\left[a_{n}\left(\theta_{1}\right),\;a_{n}\left(\theta_{2}\right),\;\cdots \;,a_{n}\left(\theta_{K}\right)\right],$$

whose columns denote array steering vectors for *K* signal sources of the *n*th frequency bin, that is

(5) $$a_{n}\left(\theta_{k}\right)={\left[e^{-2j\pi f_{n}\tau_{m}\left(\theta_{k}\right)},\;e^{-2j\pi f_{n}\tau_{2}\left(\theta_{k}\right)},\;\cdots ,\;e^{-2j\pi f_{n}\tau_{M}\left(\theta_{k}\right)}\right]}^{T},$$

where $a_{n}\left(\theta_{k}\right)\in C^{M}$, $\tau_{m}\left(\theta_{k}\right)=h_{m}sin\left(\theta_{k}\right)/c$, and *c* is the signal source propagation velocity.

Then, the covariance matrix $R_{x_{n}}$ of the *n*th bin data can be expressed as

(6) $$\begin{array}{cc} R_{x_{n}} & =E\left[x_{n,l}x_{n,l}^{H}\right]\\ \; & =A_{n}\left(\theta \right)R_{s_{n}}A_{n}{\left(\theta \right)}^{H}+R_{w_{n}}\\ \; & =A_{n}\left(\theta \right)\mathrm{diag}\left(\rho_{n}\right)A_{n}{\left(\theta \right)}^{H}+\sigma_{w}^{2}I, \end{array}$$

where $R_{s_{n}}=E\left[s_{n,l}s_{n,l}^{H}\right]=\mathrm{diag}\left(\rho_{n}\right)$ denotes the only signal covariance matrix at the frequency bin $f_{n}$, and $\rho_{n}={\left[\rho_{n,1}^{2},\;\rho_{n,2}^{2},\;\cdots ,\;\rho_{n,K}^{2}\right]}^{T}$ is the source power vector, while $R_{w_{n}}=E\left[w_{n,l}w_{n,l}^{H}\right]=\sigma_{w}^{2}I$ is the noise covariance matrix, and $\sigma_{w}^{2}={\left[\sigma_{n,1}^{2},\;\sigma_{n,2}^{2},\;\cdots ,\;\sigma_{n,M}^{2}\right]}^{T}$ represents the noise power vector.

In practice, the covariance matrix $R_{x_{n}}$ is estimated from the sample covariance matrix ${\hat{R}}_{x_{n}}$ with the *L* non-overlapping segments, and ${\hat{R}}_{x_{n}}$ is given by

(7) $${\hat{R}}_{x_{n}}=\frac{1}{L}\sum_{l=1}^{L}x_{n,l}x_{n,l}^{H},$$

Vectorizing ${\hat{R}}_{x_{n}}$ in Equation (7), we have

(8) $$r_{n}=\mathrm{vec}\left\{{\hat{R}}_{x_{n}}\right\}=B_{n}\left(\theta \right)q_{n}+\sigma_{w}^{2}\overset{ˇ}{I},$$

where

(9) $$\begin{array}{cc} B_{n}\left(\theta \right) & =A_{n}{\left(\theta \right)}^{*}⊙A_{n}\left(\theta \right)\\ \; & =\left[b_{n}\left(\theta_{1}\right),\;b_{n}\left(\theta_{2}\right),\;\cdots ,\;b_{n}\left(\theta_{K}\right)\right]\in C^{M^{2}\times K}, \end{array}$$

with

(10) $$b_{n}\left(\theta_{k}\right)=a_{n}^{*}\left(\theta_{k}\right)⊗a_{n}\left(\theta_{k}\right),$$

where $q_{n}={\left[\rho_{n,1}^{2},\;\rho_{n,2}^{2},\;\cdots ,\;\rho_{n,K}^{2}\right]}^{T}$ represents the signal vector, and $\overset{ˇ}{I}=\mathrm{vec}\left\{I\right\}\in C^{M^{2}\times 1}$. Note that $B_{n}\left(\theta \right)$ is a tall matrix and can provide useful information for increasing the DOF; in other words, $M<Rank\left(B_{n}\left(\theta \right)\right)<M^{2}$. According to the concept of difference co-array [19], we can get a new virtual array position of the ULA

(11) $$\begin{array}{cc} \bar{H} & =\{h_{q}-h_{p}:q,p=1,\;2,\;\cdots ,\;M\}\\ \; & =\left\{\left(-M_{2}\left(M_{1}+1\right)+1\right)d_{1},\;\cdots ,\;0,\;d_{1},\;\cdots ,\;\left(M_{2}\left(M_{1}+1\right)-1\right)d_{1}\right\}\in R^{1\times \tilde{M}}, \end{array}$$

where $\tilde{M}=2M_{2}\left(M_{1}+1\right)-1$.

The goal of the paper is to determine the θ from $r_{n}$ in Equation (8).

## 3. Wideband Signal Processing Using Virtual Array

### 3.1. Virtual Array Signal

Recall that the vector $r_{n}$ in Equation (8) contains the repeat rows, which do not help for improving the DOF. After removing the repeat rows and sorting its remaining rows of the $r_{n}$, we obtain a new vector by the following operation

(12) $$z_{n}=G_{n}\left(\theta \right)q_{n}+{\tilde{w}}_{n},$$

where $z_{n}\in C^{\tilde{M}}$ and ${\tilde{w}}_{n}=\sigma_{w}^{2}{\tilde{I}}_{\tilde{M}}\in C^{\tilde{M}}$ represent the new observation vector and the new noise vector, respectively. $G_{n}\left(\theta \right)=\left[g_{n}\left(\theta_{1}\right),\;g_{n}\left(\theta_{2}\right),\;\cdots ,\;g_{n}\right]\in C^{\tilde{M}\times K}$ is redefined as the filled virtual manifold matrix, which is equivalent to the counterpart of a ULA with $\tilde{M}$ sensors. The virtual array steering vector $g_{n}\left(\theta_{k}\right)$ exhibits a Vandermonde structure of size $\tilde{M}$ as follows

(13) $$g_{n}\left(\theta_{k}\right)=\left[\begin{array}{c} e^{-2j\pi f_{n}\tau_{\left(-M_{2}\left(M_{1}+1\right)+1\right)}\left(\theta_{k}\right)}\\ e^{-2j\pi f_{n}\tau_{\left(-M_{2}\left(M_{1}+1\right)+2\right)}\left(\theta_{k}\right)}\\ \vdots \\ e^{-2j\pi f_{n}\tau_{0}\left(\theta_{k}\right)}\\ \vdots \\ e^{-2j\pi f_{n}\tau_{\left(M_{2}\left(M_{1}+1\right)-1\right)}\left(\theta_{k}\right)} \end{array}\right],$$

### 3.2. Focusing on the Virtual Array

It is known that the CSM, which is a classic way of wideband signal processing [31], can lead to less complexity and better DOA estimation performance. The key point of CSM is to design focusing matrices ${\left\{P_{n}\right\}}_{n=1}^{N}$, whose purpose are to transform the manifold matrices at frequencies ${\left\{f_{n}\right\}}_{n=1}^{N}$ to that at the pre-selected reference frequency $f_{0}$ as follows [32]

(14) $$P_{n}G_{n}\left(\theta \right)=G_{0}\left(\theta \right),$$

where $G_{0}\left(\theta \right)$ denotes the virtual array manifold matrix at the referenced frequency $f_{0}$. Note that rotational signal subspace (RSS) method is a well known way of CSM [13], whose focusing matrices ${\left\{P_{n}\right\}}_{n=1}^{N}$ are gained through minimizing a Frobenius norm of the obtained virtual array manifold errors

(15) $$\begin{array}{cc} \; & \min_{P_{n}}\;\;∥G_{0}\left(\theta \right){)-P_{n}G_{n}\left(\theta \right)∥}_{F}\\ \; & \;\;\;\;s.t.\;\;\;\;\;\;P_{n}P_{n}^{H}=I, \end{array}$$

where $P_{n}$ should be a unitary matrix, yielding [33]

(16) $$P_{n}=U_{n}^{r}{\left(U_{n}^{l}\right)}^{H}.$$

We now define an excessive matrix $E=G_{n}\left(\theta \right)G_{0}{\left(\theta \right)}^{H}$, whose left and right singular vectors are defined as $U_{n}^{l}$ and $U_{n}^{r}$, respectively. Accordingly, the product of the virtual array $y_{n}$ is defined through multiplying the focusing matrix by ${\bar{z}}_{n}$

(17) $$y_{n}=P_{n}{\bar{z}}_{n}=P_{n}G_{n}\left(\theta \right)q_{n}+P_{n}{\tilde{w}}_{n}^{\circ }.$$

After focusing processing, we can reconstruct a new single wideband signal model by averaging the obtained signal of each frequency as follows

(18) $$\begin{array}{cc} \bar{y}\; & =\frac{1}{N}\sum_{n=1}^{N}y_{n}=\frac{1}{N}\sum_{n=1}^{N}P_{n}{\bar{z}}_{n}\\ \; & =\frac{1}{N}\sum_{n=1}^{N}P_{n}G_{n}\left(\theta \right)q_{n}+\frac{1}{N}\sum_{n=1}^{N}P_{n}{\tilde{w}}_{n}^{\circ }\\ \; & =G_{0}\left(\theta \right)\bar{q}+\bar{P}{\tilde{w}}_{n}^{\circ }, \end{array}$$

where $\bar{q}=\frac{1}{N}\sum_{n=1}^{N}q_{n}\in C^{K\times 1}$ is a column vector and $\bar{P}=\frac{1}{N}\sum_{n=1}^{N}P_{n}$. For the Gaussian white noise, there is ${\tilde{w}}_{n}^{\circ }=\sigma_{w}^{2}{\tilde{I}}_{\tilde{M}}$.

### 3.3. Source Number Detection Using Information Theory Criteria

As is known, the key factor for source number detection is the obtained sample covariance matrix. Unfortunately, we cannot get an optimal covariance matrix directly by the new reconstructed single wideband signal in Equation (18). The spatial smoothing method is one of effective solutions for such problem. We can divide the virtual array into $\frac{\tilde{M}+1}{2}+1$ subarrays, each with $\frac{\tilde{M}-1}{2}-1$ sensors. The output of the $n^{\prime }$ th subarray is defined as ${\bar{y}}_{n^{\prime }}$, whose corresponding covariance matrix $R_{n^{\prime }}$ is written as

(19) $$R_{n^{\prime }}={\bar{y}}_{n^{\prime }}{\bar{y}}_{n^{\prime }}^{H}.$$

Meanwhile, we can obtain the averaged covariance matrix *R*, that is

(20) $$R=\frac{1}{\left(\frac{\tilde{M}+1}{2}+1\right)}\sum_{n^{\prime }=1}^{\frac{\tilde{M}+1}{2}+1}R_{n^{\prime }}.$$

Afterwards, applying EVD on the matrix *R*, we have

(21) $$R=U\mathrm{diag}\left(\lambda \right)U^{H},$$

where U and λ are the eigen vector matrix and eigenvalues, respectively. Here, we have the non-increasing order of such eigenvalues

(22) $$\lambda_{1}\geq \lambda_{2}\geq \cdots \geq \lambda_{\frac{\tilde{M}-1}{2}-1}.$$

We next introduce the information theory methods: AIC, AIC-C3, and MDL  [29,30] for source number detection. These three methods can be organized as follows:

(23) $$AIC\left(k\right)=2\tilde{L}\left(\tilde{M}-k\right)log\Lambda \left(k\right)+2k\left(2\tilde{M}-k\right)$$

(24) $$MDL\left(k\right)=\tilde{L}\left(\tilde{M}-k\right)log\Lambda \left(k\right)+\frac{1}{2}k\left(2\tilde{M}-k\right))log\tilde{L}$$

(25) $$AIC-C3\left(k\right)=2\tilde{L}\left(\tilde{M}-k\right)log\Lambda \left(k\right)+C,$$

where Λ(k) is the maximum likelihood

(26) $$\Lambda \left(k\right)=\frac{\frac{1}{\tilde{M}-k}\sum_{i=k+1}^{\tilde{M}}\lambda_{i}}{{\left(\prod_{i=k+1}^{\tilde{M}}\lambda_{i}\right)}^{\frac{1}{\tilde{M}-k}}},$$

and

(27) $$C=\frac{\left(4\tilde{M}k-2k^{2}+2\right)\left(2\tilde{M}\tilde{L}+2\tilde{M}k+k^{2}+2\right)}{2\tilde{M}\tilde{L}-2\tilde{M}k+k^{2}-2}-\frac{4\tilde{M}k-2k^{2}}{2\tilde{M}\tilde{L}-2\tilde{M}k+k^{2}}.$$

where $\tilde{L}$ and *k* represent the number of snapshots and the number of DOF, respectively.

Then, we estimate the signal source number K by

(28) $$\begin{array}{cc} \; & {\hat{K}}_{AIC}=\arg\;\min\;AIC(k)\\ \; & {\hat{K}}_{MDL}=\arg\;\min\;MDL(k)\\ \; & {\hat{K}}_{AIC-C3}=\arg\;\min\;AIC-C3(k). \end{array}$$

### 3.4. DOA Estimation via ANM

#### 3.4.1. ANM Principle

In the literature  [28,34], the ANM principle is that the signal is expressed as a linear combination of a few atoms over a known atom set, and then, the structural information of these atoms, i.e., Vandermonde structure, is utilized for signal reconstruction from measurements.

We assume the signal Φ contains several components, which exhibit the same Vandermonde structure and especially belong to a basic atom set A. Therefore, we can define the atomic norm of Φ over the atom set A, which has the form

(29) $${\left\|\;\Phi \;\right\|}_{A}=\inf\left\{\sum_{n}\left|s_{n}\right||\Phi =\sum_{n}s_{n}A_{n},\;\;A_{n}\in A\right\},$$

whose main task is to seek the sparsest decomposition of Φ over A. In other words, if the signal Φ has a special form, i.e., a linear combination form, the signal Φ is considered to be sparse over the atom set of A in Equation (29). Knowing the A, the components of Φ can be retrieved using $\ell_{1}$ norm minimization as follows:

(30) $$\underset{A_{n},s_{n}}{\arg\min}\left\{\sum_{n}\left|s_{n}\right|\;\;\;s.t.\;\Phi =\sum_{n}s_{n}A_{n},\;\;A_{n}\in A\right\}.$$

Equation (30) can be tackled by seeking out the atomic norm ${\left\|\;\Phi \;\right\|}_{A}$, while the optimal atom set is also obtained for estimating DOAs.

In the presence of noise, the observed measurement Ψ=Φ+w contains the signal and the noise, thus the corresponding atomic norm is written as

(31) $$\min_{\Phi }{\left\|\;\Phi \;\right\|}_{A}\;\;\;\;s.t.\left\|\;\Psi -\Phi \;\right\|\;\leq \;\varepsilon ,$$

where ε denotes the noise threshold. The problem in Equation (31) is called the atomic norm minimization, which can be solved via SDP.

#### 3.4.2. DOA Estimation

Here, recall Equation (18); in the case of being noiseless, Equation (18) only contains the signals information, which can be rewritten as

(32) $$\bar{y}=s^{★}\overset{\Delta }{=}G_{0}\left(\theta \right)\bar{q}=\sum_{k=1}^{K}{\bar{q}}^{k}g_{0}\left(\theta_{k}\right),$$

where ${\bar{q}}^{k}=\frac{1}{N}\sum_{n=1}^{N}q_{n}^{k}=\frac{1}{N}\sum_{n=1}^{N}{\left(\rho_{n}^{k}\right)}^{2}$, and all *K* steering vectors ${\left\{g_{0}\left(\theta_{k}\right)\right\}}_{k=1}^{K}$ have the same structure, i.e., Vandermonde structure. Furthermore, they belong to a known atom set $A_{v}$. According to the ANM definition, the signal $s^{★}$ can be considered as the sparse signal over the atom set $A_{v}$.

Afterwards, the set of atoms by all possible $G_{0}\left(\theta \right)$ can be readily formed as follows:

(33) $$A_{v}:=\left\{G_{0}\left(\theta \right):\theta \in \left(-\pi /2,\pi /2\right]\right\}.$$

Apparently, the noiseless version of Equation (32) of $\bar{y}$ is viewed as a positive combination of complex sinusoids over the atom set $A_{v}$. It has been indicated that the atomic norm of $s^{★}$ is said to be minimized, when only those atoms corresponding to the true signals ${\left\{\theta_{k}\right\}}_{k=1}^{K}$ are selected to linearly describe $s^{★}$, which has the form

(34) $${∥s^{★}∥}_{A_{v}}\overset{\Delta }{=}\inf\left\{\sum_{n=1}^{K}\left|{\bar{q}}^{k}\right||{\bar{q}}^{k}g_{0}\left(\theta_{k}\right),g_{0}\left(\theta_{k}\right)\in A_{v}\right\}.$$

To practically solve Equation (34), an SDP formulation of $∥s^{★}{∥}_{A}$ is given as

(35) $$\begin{array}{cc} \; & \min_{u,v}\;\;\;\;\left\{\frac{1}{2}tr\left(T\left(u\right)\right)+\frac{1}{2}v\right\}\\ \; & \;\;\;\;s.t.\;\;\;\left[\begin{array}{cc} v & {\left(s^{★}\right)}^{H}\\ s^{★} & T(u) \end{array}\right]⪰0, \end{array}$$

where T(u) is a Toeplitz matrix. According to the authors of [28,35], any PSD Toeplitz matrix allows for Vandermonde decomposition. For any $\left({\left\{{\bar{q}}^{k},\theta_{k}\right\}}_{k=1}^{K},K<\tilde{M}\right)$, define $u=\sum_{n=1}^{K}\left|{\bar{q}}^{k}\right|g_{0}\left(\theta_{k}\right)$ and the Toeplitz matrix T(u) is formed from the first row of the vector $u=[u_{1},u_{2},\cdots ,u_{\tilde{M}}]$, that is

(36) $$T\left(u\right)=\left[\begin{array}{cccc} u_{1} & u_{2} & \cdots & u_{\tilde{M}}\\ {\left(u_{2}\right)}^{H} & u_{1} & \; & u_{\tilde{M}-1}\\ \vdots & \vdots & \ddots & \vdots \\ {\left(u_{\tilde{M}}\right)}^{H} & {\left(u_{\tilde{M}-1}\right)}^{H} & \cdots & u_{1} \end{array}\right].$$

The SDP yields the optimal vector u and the Toeplitz matrix T(u), in which the true signals θ are estimated.

In practice, we usually do not have the $s^{★}$ directly, and the measurement $\bar{y}$ contains the noise in Equation (18). According to Equation (31), the ANM formulation can be written as

(37) $$\min_{{\hat{s}}^{★}}\left\{\xi \;{\left\|\;{\hat{s}}^{★}\;\right\|}_{A_{v}}+{\;\|\;\bar{y}-{\hat{s}}^{★}\;\|}_{2}^{2}\right\}.$$

Equation (37) makes a regularized denoising formulation to recover $s^{★}$. $\|{\hat{s}}^{★}{\|}_{A_{v}}$ represents the sparsity enforcing term, and the quadratic term of Equation (37) is used to control the noise. ξ is a regularization parameter.

According to Equation (35), Equation (37) now can be converted to an equivalent SDP formulation as follows:

(38) $$\begin{array}{cc} \; & \min_{\hat{u},v,{\hat{s}}^{★}}\;\;\;\;\left\{\frac{1}{2}\left(tr\left(T\left(\hat{u}\right)\right)+v\right)+\xi {∥\bar{y}-{\hat{s}}^{★}∥}_{2}^{2}\right\}\\ \; & \;\;\;\;s.t.\;\;\;\;\;\left[\begin{array}{cc} v & {\left({\hat{s}}^{★}\right)}^{H}\\ {\hat{s}}^{★} & T(\hat{u}) \end{array}\right]⪰0, \end{array}$$

where ξ is a regularization parameter balancing Toeplitz structure and the noise tolerance to the observation $\bar{y}$, i.e., $∥\bar{y}-{\hat{s}}^{★}{∥}_{2}^{2}$ (least-squares term), whose poor choice may increases the probability of a bad solution. Therefore, an optimal section of ξ plays a very important role in guaranteeing signal recover. The SDP formulation in Equations (35) and (38) can be implemented efficiently by the CVX toolboxes.

After attaining the optimal Toeplitz matrix $T(\hat{u})$ in Equation (38), the subspace Root-MUSIC method is utilized to estimate the direction of *K* signal sources, $\hat{\theta }=\left[{\hat{\theta }}_{1},\;{\hat{\theta }}_{2},\;\cdots \;,{\hat{\theta }}_{K}\right]$.

*Remark on targets angle separation condition*: Here, targets angle separation means the required angle separation between neighbouring signal source. According to the IV.2 theorem [34], the sufficient separation condition is

(39) $$\begin{array}{cc} \; & sin\left(▵\theta_{min}\right)\geq \frac{1}{\left\lfloor \frac{\left(\tilde{M}-1\right)}{4}\right\rfloor }\\ \; & ▵\theta_{min}\geq arcsin\left(\frac{1}{\left\lfloor \frac{\left(\tilde{M}-1\right)}{4}\right\rfloor }\right), \end{array}$$

Note that Equation (39) is only a sufficient condition in ANM, and always much more conservative in most realistic applications. That is to say, if Equation (39) is indeed not satisfied, one may have a chance to get the DOAs.

The overall implementation of our proposed solution is summarized as Algorithm 1.
**Algorithm 1** The proposed undetermined wideband DOA estimation method.**Input:**   The data: X;**Output:**
   Estimation of $\theta :\hat{\theta }$; 
1: Wideband signal processing and obtain the output of the frequency domain after DFT: $x_{n,l}$;2: Obtain the covariance matrix of each frequency: $R_{x_{n}}$;3: Vectorizing: $r_{n}=\mathrm{vec}\left\{R_{x_{n}}\right\}$;4: Reduce redundancy and sort by (12);5: Fix the focusing frequency: $f_{0}$;6: Calculate the focusing matrices: $P_{1},\;P_{2},\;\cdots ,\;P_{N}$ by (15) and (16);7: Recover the optimal Toeplitz matrix: $T(\hat{u})$ through SDP via (38);8: Estimate the DOAs via Root-MUSIC.


## 4. Simulation Results

To show the benefits of our proposed method, we used several simulations to testify its better DOA estimation performance, compared to the existing wideband DOA estimation methods: W-SpSF method [18], spatial smoothing MUSIC (SS-MUSIC) [36], and OGSBL [37]. Moreover, to better demonstrate the estimation performance of these four methods, the Cramer–Rao lower bound (CRLB) for wideband signal provided in [18,38] was used as the benchmark, which represents a lower bound for unbiased estimator. In addition, we used the information theory methods, i.e., AIC, MDL, and AIC-C3, to detect the signal source number.

### 4.1. Simulation Setting

**Figure 2:**
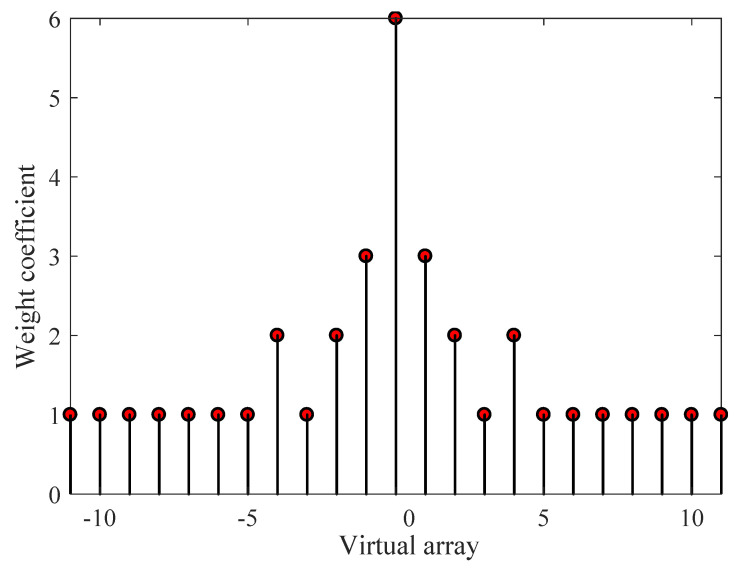
Weight coefficient of co-array of NA with six sensors.

We considered a two-level nest array with six physical sensors, whose inner sub-array and outer sub-array have the same amount of sensors, that is to say, $M_{1}$ is equal to $M_{2}=3$. The physical sensors locate at $\left[0,\;d_{1},\;2d_{1},\;3d_{1},\;7d_{1},\;11d_{1}\right]$, and $d_{1}$ is equal to 1/2λ, where λ represents the wavelength corresponding to the center frequency $f_{0}=\left(f_{l}+f_{h}\right)/2$. In addition, c=1500 m/s denotes the signal propagation velocity of underwater acoustic scenarios. Therefore, we have $d_{1}=l/2\lambda =c/\left(2f_{0}\right)$. The unchangeable default simulation parameters setting is shown in Table 1, and a co-array of NA with six sensors is displayed in Figure 2.

### 4.2. Performance Comparison

#### 4.2.1. Source Number Detection

**Figure 3:**
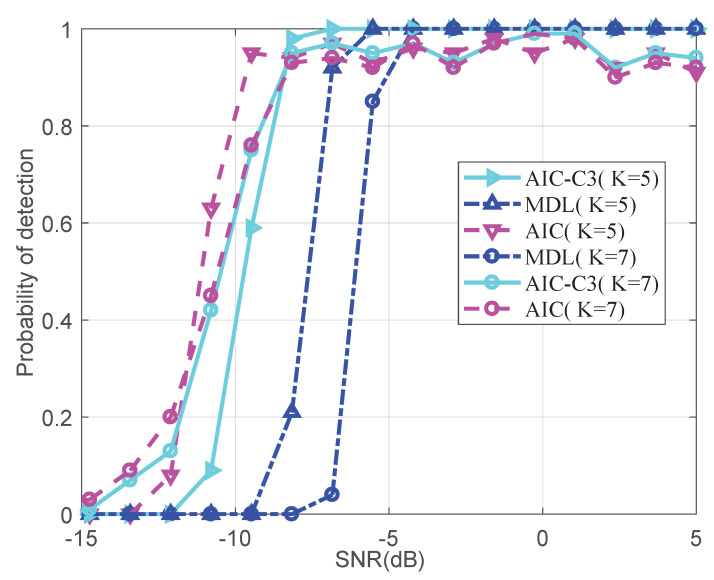
Probability of detection versus SNRs.

To verify the detection accuracy of wideband signal with NA, we compared the probability of detection using the mentioned information theory method in the overdetermined and underdetermined cases, respectively. Under the overdetermined scenario, we assumed that five wideband signals impinge on the NA from $-30^{\circ },\;-15^{\circ },\;0^{\circ },\;15^{\circ },\;29^{\circ }$, and seven wideband signal sources are from $-45^{\circ },-30^{\circ },\;-15^{\circ },\;0^{\circ },\;15^{\circ },\;29^{\circ },\;44^{\circ }$ for the underdetermined scenario. We varied the SNR from −15 to 5 dB, and fixed snapshots to 30. Figure 3 shows the result of the probability of detection versus SNR. The detection accuracies of AIC are the highest in low SNR, followed by AIC-C3 and MDL, as the case may be. The detection accuracies of all information theory methods for five signals are higher than those of seven signals. Table 2 displays the needed SNRs of the three methods, when the probability of detection are equal to 0.9 and 1, respectively.

#### 4.2.2. DOA Estimation

**Figure 4:**
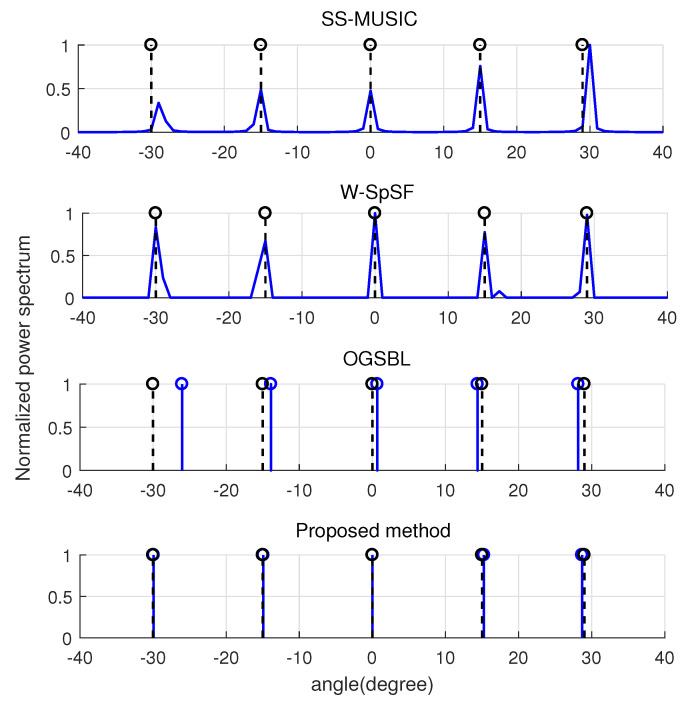
Normalized spatial spectrum for K=5 sources when M=6, L=30, and SNR=−5 dB.

We compared the normalized spatial spectrum of all methods under the overdetermined and underdetermined scenarios, respectively. Firstly, five uncorrelated signal sources were at $\left[-30^{\circ },\;-15^{\circ },\;0^{\circ },\;15^{\circ },\;29^{\circ }\right]$ and the SNR and snapshots were set as −5 dB and 30, respectively. In addition, the grid size was 0.5 degree in W-SpSF method. Figure 4 displays the normalized spatial spectrum for the five signal sources with the NA of six sensors. In Figure 4, the dashed lines are used to represent the true signal sources, while the solid lines stand for the estimated DOAs. It can be seen that five wideband signals can be detected successfully by SS-MUSIC, W-SpSF method, OGSBL, and the proposed method in the low SNR. However, there is a sidelobe peak with the performance of W-SpSF, and worse accuracy for some angles are displayed with the performance SS-MUSIC and OGSBL. Therefore, our proposed method has the best detection and estimation performance as the DOA estimations are closest to the true signals.

**Figure 5:**
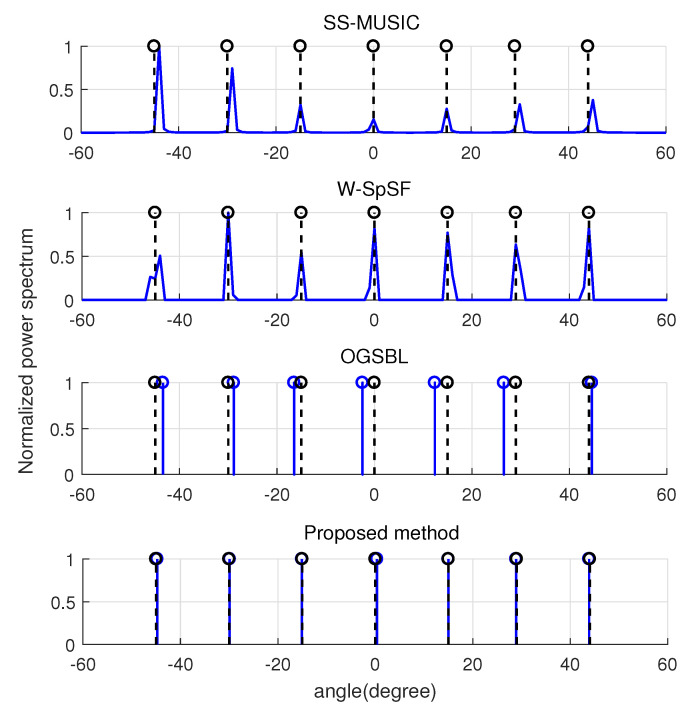
Normalized spatial spectrum for K=7 sources when M=6, L=30, and SNR=−5 dB.

Then, we compared the detection performance of all methods for the underdetermined case. The seven signals were the same as in Figure 3. The SNR and snapshots were again fixed as −5 dB and 30, respectively. Figure 5 shows the normalized spatial spectrum for the five signal sources of all methods, respectively. It is shown that all methodd can detect seven peaks successfully in the normalized spatial spectrum nearby the true DOAs. The normalized power of SS-MUSIC is worse for some signals, and there is a certain deviation in this method. W-SpSF method has a sidelobe and a certain deviation, as well. Although OGSBL has stronger normalized power, its DOA estimations are farthest from the true signals. Figure 5 shows that the propose method gives the best detectable capability, since it has strong normalized power and its estimated DOAs are closest to the true signals.

**Figure 6:**
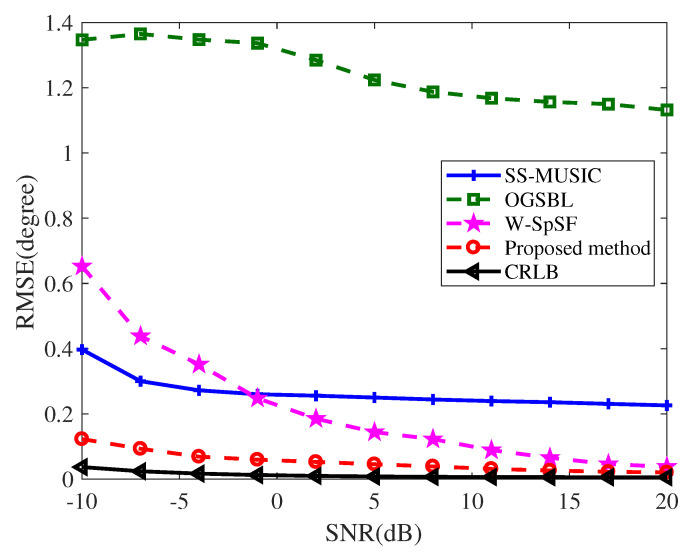
RMSE of DOA estimation for K=7 sources versus SNRs when M=6 and L=30.

Afterwards, to evaluate the estimation accuracy of our proposed method, we give the estimation results of all methods in the light of the root mean square error (RMSE), that is

(40) $$\mathrm{RMSE}=\sqrt{\frac{1}{KQ}\sum_{k=1}^{K}\sum_{q=1}^{Q}{\left({\hat{\theta }}_{kq}-\theta_{k}\right)}^{2}}.$$

where $\theta_{k}$ and ${\hat{\theta }}_{kq}$ denote the *k*th true angle and the *k*th estimated angle in the *q*th Monte Carlo trial, respectively. Unless otherwise specified, seven wideband signals impinged on the NA from $-45^{\circ },-30^{\circ },\;-15^{\circ },\;0^{\circ },\;15^{\circ },\;29^{\circ },\;44^{\circ }$. We varied SNR from −10 to 20 dB at the step of 3 dB and fixed snapshots to L=30. Figure 6 shows the results averaged over Q=100 Monte Carlo trials as a function of SNR. It can be observed that OGSBL and SS-MUSCI have the same performance, whose estimation accuracies do not improve markedly with the SNR increasing. Although the W-SpSF method outperforms the SS-MUSIC method when SNR>−1 dB, and its RMSE curve is gradually close to the CRLB curve, our proposed method’s estimation accuracy is the closest to the counterpart of CRLB. The reason is that the method we propose can recover the optimal covariance matrix with Toeplitz structure, which is beneficial to obtain accurate DOAs.

**Figure 7:**
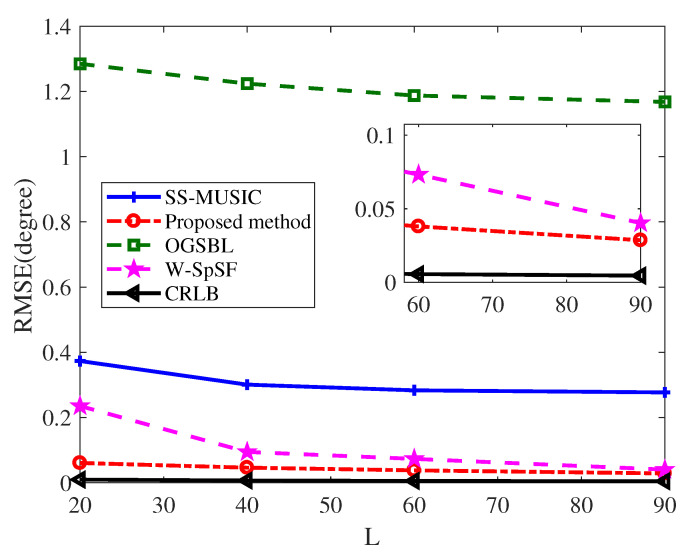
RMSE of DOA estimation for K=7 sources versus the number of snapshots when M=6 and SNR=5 dB.

Finally, to further illustrate the estimation performance of our proposed method, the RMSE versus the number of snapshots was examined for all methods. In this simulation, the value of snapshots was varied from 20 to 90, while the SNR was fixed to 5 dB. In Figure 7, we note that all methods can obtain more accurate estimation performance by increasing the number of snapshots. Obviously, the RMSE of OGSBL is still the largest among all methods followed the RMSE of SS-MUSIC (Figure 7). Although the W-SpSF RMSE curve tends to decrease most quickly versus the number of snapshots, its performance is worse than the counterpart of the proposed method in all regions. Therefore, under the case of same snapshots, the proposed method exhibits better performance in comparison with SS-MUSIC, OGSBL, and W-SpSF method.

## 5. Conclusions

In this paper, a novel wideband DOA estimation technique equipped with NA is proposed, which seeks and utilizes the Toeplitz structure of the reconstructed virtual array by ANM and thus can work well under the underdetermined wideband case. Our method provides an effective solution through collecting the vectorized covariance matrix via sub-band focusing operation to reformulate the single wideband model of virtual array, recovering the optimal Toeplitz covariance matrix and improving the Root-MUSIC by Toeplitz structure enhancement. Numerical simulations showed that the proposed method outperforms the state-of-the-art methods. In the next work, we will continue to study the wideband coherent signals DOA estimation via ANM with sensors position error.

## Figures and Tables

**Table 1:** The default simulation settings.

Parameter	Value
Number of sensors (M)	6
Number of sensors of inner sub-array $\left(M_{1}\right)$	3
Number of sensors of outer sub-array $\left(M_{2}\right)$	3
Low frequency $\left(f_{l}\right)$	1000 Hz
High frequency $\left(f_{h}\right)$	5000 Hz
Center frequency $\left(f_{0}\right)$	3000 Hz
Velocity of underwater acoustic (c)	1500 m/s
Azimuthal range	0–180 degree

**Table 2:** The probability of detection of the mentioned methods versus SNR.

Methods	*Pd* = 0.9		*Pd* = 1	
	Overdetermined Case	Underdetermined Case	Overdetermined Case	Underdetermined Case
AIC	−10 dB	−8.2 dB	\	\
AIC-C3	−8.2 dB	−8.2 dB	−6.8 dB	1.0 dB
MDL	−6.8 dB	−5.0 dB	−5.5 dB	−4.2 dB
